# Unified Anatomical Explanation of Diagonal Earlobe Creases, Preauricular Creases, and Paired Creases of the Helix

**DOI:** 10.7759/cureus.27929

**Published:** 2022-08-12

**Authors:** Mohammed Abrahim

**Affiliations:** 1 Family Medicine, McMaster University, Hamilton, CAN

**Keywords:** anterior tragal line, buccal fat pad, pcos and metabolic syndrome, metabolic syndrome (mets), facial fat, visceral adiposity, visceral fat depot, preauricular creases, diagonal earlobe crease. frank’s sign, coronary artery disease

## Abstract

Three types of ear creases have been described in the medical literature in association with several cardiovascular and metabolic disorders: diagonal earlobe creases (DELCs) (Frank’s sign), preauricular vertical creases, and paired ear creases of the helix (PECH). The exact mechanism of development of such creases, as well as an explanation of their association with cardiometabolic disorders, remains unclear. Herein, the author provides a suggested unified mechanism of development of all three types of auricular creases. In addition, an anatomical explanation linking the associated cardiometabolic disorders and the three types of ear creases to the common culprit of facial visceral adiposity will be given.

## Introduction

Globally, cardiovascular disease and its associated metabolic disorders are the leading cause of mortality [1]. Physical examination signs remain credible diagnostic indicators of cardiovascular and metabolic disorders. Three types of ear creases have been described in the medical literature with evidence of their associations to various cardiometabolic disorders, including heart disease, stroke, and type 2 diabetes. The first described, and most studied, type of ear crease is the diagonal earlobe crease (DELC), also known as Frank's sign [2]. Secondly, vertical creases anterior to the tragus, a single crease is termed anterior tragal line whereas multiple creases are termed preauricular vertical creases [3]. Finally, two creases located at the upper pole of the ear helix are known as the paired ear creases of the helix (PECH) [4]. It is essential to make a clear distinction between skin creases and skin folds. Skin creases are permanent and irreversible lines in the skin that develop secondary to prolonged traction by an underlying attachment to the deep structures whereas skin folds are created by skin redundancy and are not necessarily permanent [5].

The exact mechanism of development of such ear creases and their connection to cardiovascular disorders remain unclear. In this brief technical report, the author provides an anatomical explanation of the mechanism of development of ear creases and the likely cause of their link to cardiometabolic disorders via facial visceral obesity.

Obesity, particularly, visceral obesity is an established risk factor for cardiovascular and metabolic disorders with a growing body of research demonstrating a potential causal association independent of total body weight [6]. Visceral fat depots include intra- and inter-organ fat inside the abdominal and thoracic cavities in addition to the head [7]. Despite being anatomically separate, the buccal fat pad (BFP) of the cheeks and abdominal visceral adipose tissue appear to be metabolically and histologically identical [7]. Furthermore, the size of the BFP was strongly corresponding with the size of abdominal visceral fat even in normal-weight individuals [8]. Moreover, in overweight adults, the size of the BFP, obtained from ultrasound measurements, was found to be significantly correlated with all anthropometric parameters, including total body weight and body mass index (BMI) [9]. The distance between both inferior earlobes is the strongest predictor of the size of visceral dispose tissue [10]. Interestingly, when using serial MRI scanning of lateral facial fat compartments, the same patients revealed an increase in the size of the BFP with aging [11]. The earliest study suggesting an obesity association with DELC was in 1977 [12]. The population-based study of coronary heart disease, among 1,237 American Japanese men aged 50-74, found that “creases are prevalent in fat men” without significant correlation with coronary artery disease (CAD) [12]. In January of 2021, the author of the current article reported an association between visceral obesity of the BFP and premature coronary disease and termed the condition “sideburns obesity syndrome” [13].

## Technical report

Anatomical background

**Figure 1:**
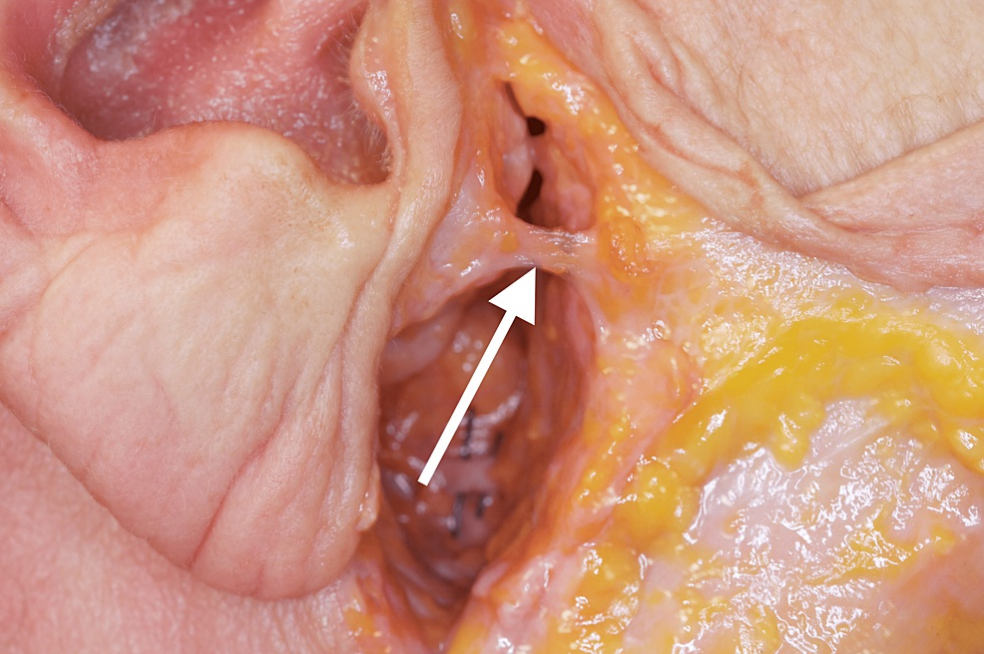
Cadaver dissection demonstrating the attachment of Loré’s fascia into the intertragal incisura of the auricle (Courtesy: Dr. Justin X. O’Brien).

The BFP is a deep encapsulated biconvex mass of adipose tissue located between the masticatory muscles within the lateral aspect of the face which was first described in 1802 by the French anatomist Xavier Bichat [14]. The exact anatomy of the facial fat compartments was recently and precisely described and BFP is referred to as the visceral fat of the face [7]. Lateral to the BFP lies the parotid gland which is attached by the tympanoparotid fascia as a band running from the parotid gland to the intertragal incisura of the auricular cartilage to the depth of the tympanomastoid fissure of the skull [15]. Through dissection, Hwang et al. described the tympanoparotid ligament’s insertion as two-thirds into the tympanomastoid fissure and one-third from the auricular cartilage and interfused with the parotid fascia, covering the parotid gland in front of the tragus after cadaver dissection [16]. They further demonstrated that Loré’s ligament is present in 100% of the investigated bodies. Functionally, Loré’s fascia is a retaining ligament [17]. The retaining ligaments of the face are necessarily strong and fixate facial soft tissues to key bony landmarks. Due to its significant strength, the ligament is utilized as a hook during facelift surgery [18]. A rare image of the dissected Lore’s ligament is demonstrated herein (Figure 1).

Anatomical explanation

In visceral obesity, the size of deep cheek fat of the BFP increases, anchoring over its fixed attachment of Lore’s ligament to the skull. This results in skin redundancy of the cheek that causes pleating of the skin in front of the ear creating the anterior tragal line and preauricular vertical creases. The author views preauricular creases as skin folds rather than true permanent creases. Furthermore, traction at the base of the earlobe’s attachment and folding of the earlobe lead to creasing. Over years of traction, the histopathological changes are set permanently, creating the DELC known as Frank’s sign.

With increasing facial visceral fat deposition, further auricular traction takes place. Because of this, the auricle is drawn in towards the point of Lore’s ligament anchorage at the base of the tragus. Such inward tension causes the internal collapse of the rigid cartilaginous helix which, in turn, leads to creasing at its weakest points and creates the paired creases of the helix (Video 1).

**Video 1:** Anatomical explanation of the three ear creases and their association to cardiovascular diseases.

## Discussion

**Figure 2:**
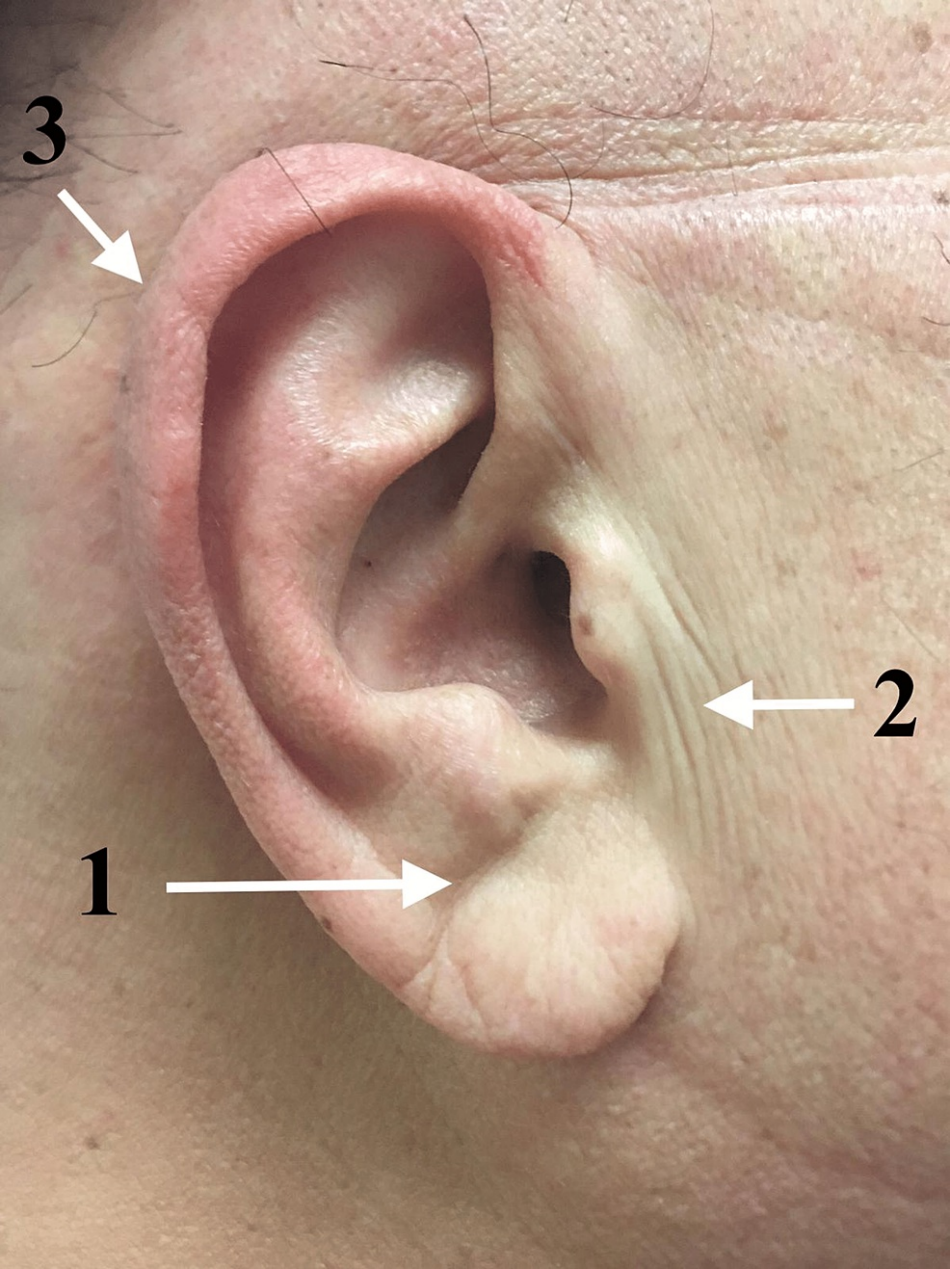
1. Diagonal earlobe crease. 2) Preauricular vertical crease. 3) Crease of the helix.

After presenting our hypothesis on the mechanism of development of the reported three ear creases: a DELC (Figure 2: point 1); preauricular vertical creases (Figure 2: point 2); and PECH (Figure 2: point 3), it is prudent to explore some previously presented and common hypotheses forwarded in this field of inquiry.

Previous hypotheses

Frank’s sign (DELC) has been extensively studied more than the other auricular creases. Although DELC was described 50 years ago, it has been traced back thousands of years. The most ancient evidence of Frank’s sign dates back to several busts and sculptures of the Roman emperor Hadrian (76-138 AD) clearly demonstrating bilateral DELC and it was thought that he died of complications of heart disease [19]. Additionally, DELC was reported and observed in a 15th-century Italian portrait of Cardinal Ludovico Trevisan who died of symptoms suggestive of heart failure [20].

The prevalence of DELC increases with age, with the latter hypothesized as an explanation for the reported associations between DELC and cardiometabolic disorders and simply, could be a sign of aging skin [21]. DELC was also associated with decreased cognitive function which, as above, might be explained by the increasing prevalence of DELC and cognitive decline as we age [22]. Given that DELC was reported in younger adults with CAD, premature aging was then implied as an explanation -- increased vascular age in the presence of DELC and loss of dermal and vascular elastic fibers [23]. DELC is also associated with increased and premature telomere shortening leading to an accelerated aging process among Japanese patients with metabolic syndromes when compared with patients with metabolic syndrome without DELC [24]. The author of the current article has previously reported the case of an earlobe crease in a young 20-year-old female who was found to have DELC associated with metabolic syndrome and polycystic ovarian syndrome (PCOS) [25].

Arterial myoelastofibrosis, Wallerian-like degeneration in peripheral nerves, and deep-tissue fibrosis found in the base of the crease showed a significant correlation between the morphological changes in the myocardium and the presence of the ear lobe crease [26]. The origin of both DELC and cardiovascular disease could be secondary to diffuse atherosclerosis and endothelial dysfunction [27]. External mechanical bending of the auricle was suggested as a cause for all auricular creases that are secondary to auricular compression during sleep (i.e., between the skull and a hard pillow/ mattress) [4, 28]. DELC development was hypothesized as a genetically determined characteristic that takes years to manifest [29]. Decreased macrophage receptor activity, due to its involvement in atherosclerosis, has also been hypothesized as pathogenesis for the development of DELC. Since earlobe collagen and scavenger macrophage receptors are structurally similar, the inability of the body to maintain earlobe collagen would also indicate an inability to maintain the chemically similar macrophage receptor [30].

Away from conventional medicine, traditional and Chinese medicine has attempted to provide other hypotheses to explain the reported associations. According to the acupuncture somatotopic map, the area of the earlobe crease corresponds to the heart region of the auricle [31]. Furthermore, electrical skin resistance was detected among patients with CAD on the “Heart” region of the ears on the “Chinese Standard Ear-Acupoint Chart,” which was lower than the community control group of Chinese participants [32].

Special considerations

We consider preauricular creases are misnomers due to their nature as skin folds, therefore, the authors have previously suggested that preauricular folds are caused by facial visceral adiposity leading to skin pleating [33].

Earlobe creases are more prevalent in men than women, however, prevalence is increased among postmenopausal women compared with those who are premenopausal [34]. In women, facial fat distribution changes following the menopausal transition mimicking that of men [35]. Furthermore, the higher prevalence of DELC in men compared with women could be explained by the prevalence of central obesity with its associated facial obesity in men [35].

Surgical excision of bilateral BFP is termed “bichectomy” and has been gaining popularity worldwide for esthetic and reconstructive purposes [36]. The author proposes that performing a bichectomy, before skin creasing, could interfere with or prevent the occurrence of ear creases. Earlobe creasing is dependent on earlobe shape, therefore, DELC is more prevalent in free lobes than in attached lobes [37]. The less commonly soldered earlobes showed no creasing whereas free earlobes are the most common shape [37].

Ear creases could be viewed as signs of either a current or past history of long-standing visceral facial obesity as ear creases are irreversible skin creases whereas visceral obesity and its associated cardiometabolic disorders are potentially reversible. The author proposes that the term preauricular creases is a misnomer, and since they are created by skin redundancy they fit the definition of “folds” rather than “creases” as they are not fixed (non-permanent) and created by skin pleating and redundancy [38].

## Conclusions

In the current article, the author proposes that facial visceral obesity, particularly in the sideburn area of the cheek, is the common driver that explains the link between all types of ear creases and cardiometabolic disorders. It is hypothesized that the presence of auricular creases could be evidence of long-standing facial visceral obesity.
